# Cardiac Stunning in Acute Noninsular Ischemic Stroke: An Uncommon But Often Unrecognized Scenario

**DOI:** 10.1016/j.jaccas.2025.104024

**Published:** 2025-07-09

**Authors:** Jiangyong Min, Blake Senay, Asad Ahrar, Brian Wideman, Ryan Stumpo, Juan Fernando Ortiz, Nagib Chalfoun, Tia Chakraborty

**Affiliations:** a Department of Neurosciences and Comprehensive Stroke Center, Corewell Health West, Grand Rapids, Michigan, USA; b Department of Neurology, Michigan State University College of Human Medicine, Grand Rapids, Michigan, USA; c Division of Neurocritical Care, Corewell Health West, Grand Rapids, Michigan, USA; d Meijer Heart and Vascular Institute and Cardiovascular Medicine, Corewell Health West, Grand Rapids, Michigan, USA

**Keywords:** acute cerebellar stroke, cardiac stunning, neurogenic cardiac dysfunction

## Abstract

**Background:**

The negative cardiac outcome in patients with acute stroke remains inconclusive and mixed. No data were reported on neurogenic cardiac insult in patients with acute noninsular ischemic stroke, specifically cerebellar stroke, and its impact on cardiac function.

**Methods and Results:**

We reported that 4 patients who were healthy at baseline developed transient cardiac stunning after acute ischemic cerebellar stroke. Patients’ electrocardiograms and cardiac enzyme levels were normal, and no cardiac intervention was performed because of the presumed neurogenic cardiac outcome. Their cardiac dysfunction spontaneously returned to normal range within 2 to 3 months, as demonstrated by repeated cardiac magnetic resonance imaging or echocardiography.

**Conclusions:**

Although acute stroke involving the insular cortex is a common cause of cardiac stunning, strokes of other territories, such as cerebellar stroke, as shown in this case series report, can also cause neurogenic cardiac dysfunction and should not be overlooked.

## Graphical Abstract

**Figure:**
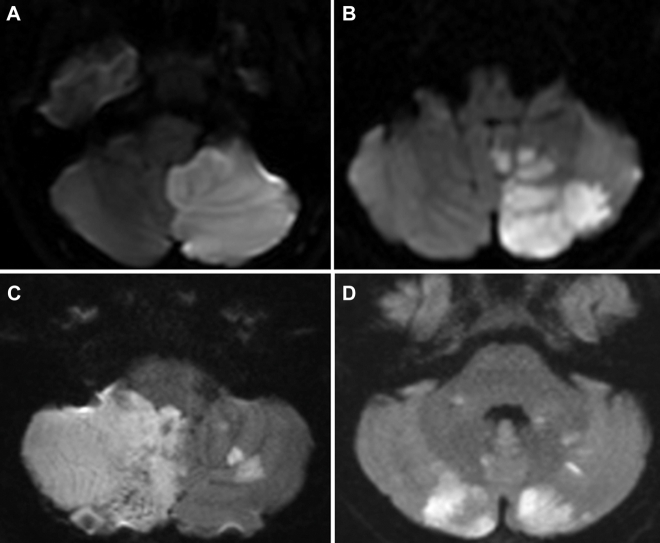

Stroke, a leading cause of neurologic death, is the leading cause of disability in the United States. Ischemic stroke represents about 85% of all strokes and is a heterogeneous condition. Negative cardiac outcomes after intraparenchymal or subarachnoid hemorrhage have been demonstrated by a large number of studies and clinical observations. Few studies have shown that cardiac arrhythmias can occur after cerebral ischemia.^1^^,^^2^ The insular cortex has attracted much attention as the possible cerebral locus for stroke-associated cardiac arrhythmia.^2^, ^3^, ^4^ Our previous animal study and recent clinical observations revealed that stroke involving the left insular cortex can result in cardiac dysfunction.^2^^,^^4^ However, the adverse cardiac outcome in patients with acute stroke remains inconclusive and mixed. Although a previous case report^5^ and a review article^6^ described that noninsular cerebellar stroke could also lead to myocardial stunning, its specific mechanism was not discussed in detail. The present case series report demonstrates a reversible phenomenon of neurogenic cardiac stunning after noninsular ischemic stroke, specifically cerebellar stroke, and its potential mechanism.

**Take-Home Messages**

• Although acute stroke involving the insular cortex is a common cause of cardiac stunning, strokes in other territories can also cause neurogenic cardiac dysfunction.
• It should not be overlooked, as demonstrated by acute cerebellar stroke in this case series report.
• Potential underlying occult cardiac pathology should be carefully evaluated, especially in the young population with acute ischemic stroke.

## Clinical Cases

### Case 1

**Figure 1:**
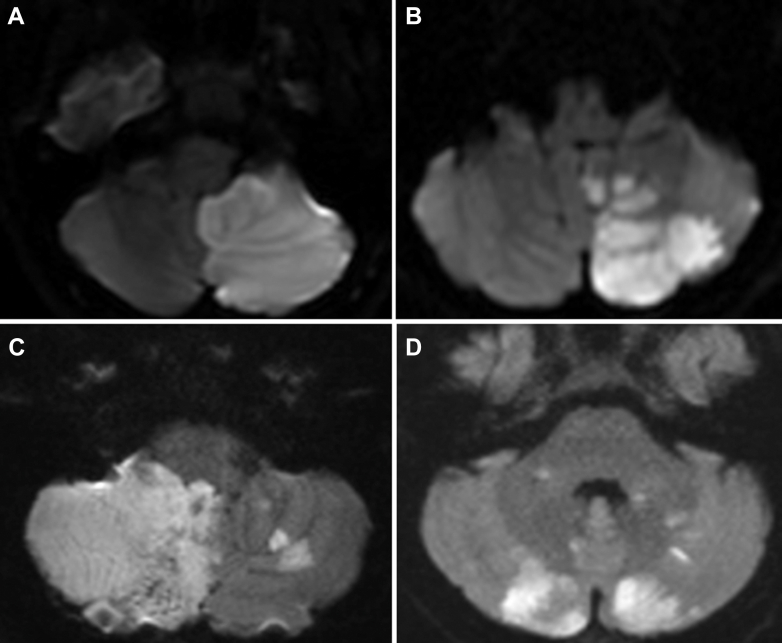
Brain MRI DWI (A) Brain MRI DWI of case 1 revealed a large left cerebellar ischemic stroke. (B) Brain MRI DWI of case 2 showed a large left cerebellar ischemic stroke. (C) Brain MRI DWI of case 3 showed a large right and small left cerebellar ischemic stroke. (D) Brain MRI DWI of case 4 showed multifocal ischemic stroke involving bilateral cerebellar hemispheres. DWI = diffuse weighted imaging; MRI = magnetic resonance imaging.

A right-handed 22-year-old Caucasian woman with a history of episodic migraine who woke up with a dull headache, blurred vision, nausea, and vomiting was brought to an outside hospital emergency department (ED) by her family. Her vital signs during the ED course were normal, and physical examination did not reveal localized neurologic deficits. She received an intravenous (IV) infusion of migraine “cocktail” (promethazine, valproic acid, and magnesium sulfate). Her symptoms of dull headache and dizziness improved, and she was then discharged home from the ED. Her unspecified dizziness without vertigo, blurred vision, and nausea reoccurred and had worsened the following day. She was brought to the local hospital again. Upon ED arrival, physical examination revealed mild ataxia with a National Institute Health Stroke Scale (NIHSS) score of 1. An IV thrombolytic agent was not administered because the patient was outside the treatment window. Electrocardiography (ECG) showed normal sinus rhythm. Urgent brain magnetic resonance imaging (MRI) without contrast revealed a large left cerebellar stroke (Figure 1A). Computed tomography (CT) angiography of the head and neck demonstrated left vertebral artery dissection with segments of high-grade stenosis. An emergent suboccipital craniectomy was performed on day 2 because of a clinically decreased level of consciousness and worsening cerebral edema, represented by effacement of the circum-mesencephalic cistern on repeated head CT, with suspicion of imminent cerebellar herniation. Transthoracic echocardiography (TTE) was performed the following day, which showed 1) left ventricular (LV) function severely reduced, with an estimated LV ejection fraction (LVEF) of 26%; and 2) deep trabeculations noted in the LV apex. Findings were suggestive of LV noncompaction cardiomyopathy. No ventricular mass or thrombus was visualized. Cardiac enzyme levels were within the normal range. At discharge on day 7 of admission, her limb ataxia was nearly resolved. Her neurology follow-up visit 2 months later at the stroke clinic showed no residual neurologic deficits on physical examination. Cardiac MRI was performed 3 months later, which showed 1) normal cardiac function, with a measured LVEF of 57% and normal LV chamber size; and 2) extensive trabeculations in the LV, which would support a diagnosis of LV noncompaction.

### Case 2

A right-handed 63-year-old Hispanic man without baseline medical comorbidity presented to the hospital ED with vertigo and imbalance. Physical examination found left arm ataxia with an NIHSS score of 1. CT angiography was normal, with no evidence of large vessel occlusion or hemodynamic stenosis. The patient was outside the thrombolytic therapeutic window. Brain MRI the following day revealed a large left cerebellar ischemic stroke (Figure 1B). TTE showed mild to moderate global hypokinesis of the LV with an LVEF of 37% and no cardiac structural abnormality. ECG showed normal sinus rhythm without a change in ST segments or T waves. His cardiac enzymes, hypercoagulable panel, and urinary drug screening were negative. Thirty days of cardiac monitoring was unremarkable, with no evidence of atrial fibrillation. A prolonged insertable cardiac monitoring device was implanted before his hospital discharge; the stroke etiology remains cryptogenic. Repeated echocardiography 2 months later, after the index ischemic stroke, showed that the LVEF was 55% with no evidence of cardiac hypokinesis. He had returned to baseline, with no focal neurologic deficits.

### Case 3

A right-handed 19-year-old Caucasian woman with a history of anxiety, depression, and asthma and a family history of Ehlers-Danlos syndrome presented to the outside hospital ED with a headache; her neurologic examination was normal with an NIHSS score of 0. She was discharged home after giving symptomatic treatment with a migraine cocktail. Two days later, the patient developed imbalance, agitation, confusion, and delirium. She was taken to our hospital ED for further evaluation. An IV thrombolytic agent was not administered because the patient was outside the treatment window. Noncontrast head CT detected a large right and a small left cerebellar ischemic stroke with mass effect on the fourth ventricle and inferior brainstem (Figure 1C). CT venography was negative for cerebral venous sinus thrombosis. CT angiography of the head and neck revealed right distal vertebral artery dissection and occlusion. The patient was intubated in the ED because of respiratory distress and underwent a decompressive suboccipital craniotomy. Her hypercoagulable panel and urinary drug screening were negative; cardiac enzyme high-sensitivity troponin T and B natriuretic peptide levels were mildly elevated. ECG showed sinus tachycardia with no changes in ST segments or T waves. TTE was performed on the day of admission, showing an LVEF of 15% with global systolic dysfunction. No cardiac thrombus was detected. Five days later, repeated TTE revealed that the LVEF was 50%, with borderline global hypokinesis of the LV. Her repeated CT angiography 5 months later revealed occlusion of the right distal vertebral artery with short segment reconstitution of the V_3_ segment extending intracranially.

### Case 4

A right-handed 35-year-old Caucasian woman with no significant medical history presented to the hospital ED with sudden-onset neck pain, occipital headache, left-sided ataxia, and vertigo. The neurologic examination revealed left-sided weakness and ataxia with an NIHSS score of 4. IV tenecteplase was administered in the ED after negative noncontract head CT, which showed no brain hemorrhage. Initial CT angiography of the head and neck showed complete occlusion of the left vertebral artery in the distal first (V_1_) and proximal secondary (V_2_) segments, concerning acute dissection of the left vertebral artery. The patient’s neurologic condition deteriorated rapidly within about 2 hours of ED arrival, becoming obtunded and experiencing respiratory distress. Cardiac enzyme troponin T and B natriuretic peptide levels were mildly elevated. ECG showed newly developed atrial fibrillation. The patient was intubated in the ED, and repeated advanced imaging was performed, including CT angiography, which demonstrated no evidence of hemorrhage but bilateral vertebral artery occlusions concerning dissection, as well as a noted basilar artery tip thrombus. She had undergone an emergent mechanical thrombectomy for basilar artery occlusion with thrombolysis in cerebral stroke 3 reperfusion. Brain MRI (Figure 1D) revealed multifocal acute ischemic stroke within the left cerebellum, greater than the right cerebellum, midbrain, pons, and left inferior cerebellar peduncle. She received a suboccipital craniotomy because of emerging evidence of hydrocephalus clinically and radiographically. TTE was performed on day 1 of her admission, which detected a suspected nonmobile mass with an LVEF of 15% and severe global hypokinesis, with basal to mid-inferior and inferolateral akinesis. Subsequent transesophageal echocardiography was performed the following day, showing a severely dilated LV with severely reduced LV function but no LV thrombus. Repeated TTE 5 days after stroke onset revealed an improvement in cardiac function, with the LVEF increasing from the previous 15% to 30%. The basal to mid-anterior/anterolateral and mid-inferolateral segments are severely hypokinetic, with no evidence of LV thrombus. Unfortunately, the patient had unexpected spontaneous tracheal hemorrhage and cardiac arrest with unsuccessful resuscitation. She passed away on day 15 of admission.

## Discussion

Neurogenic stunned myocardium, also called neurogenic stress cardiomyopathy,^6^ is a phenomenon that can occur after severe acute neurologic injury in acute stroke involving the insular cortex.^2^, ^3^, ^4^ The underlying mechanism of this transient myocardial stunning could be secondary to autonomic dysfunction after severe cerebral insult. Our previous animal study^4^ demonstrated that myocardial levels of norepinephrine were elevated after acute left middle cerebral artery stroke involving the insular cortex, suggesting that catecholamines (CAs) may mediate cardiac dysfunction. To our knowledge, reversible transient heart failure secondary to cerebellar stroke has not been well reported in the literature.

Considerable clinical and laboratory evidence^2^^,^^4^^,^^7^^,^^8^ implicates that the disturbance of the sympathetic/parasympathetic balance secondary to insular cortex damage could result in a CA surge, which would trigger myocytolysis and subsequent cardiac dysfunction. Furthermore, intracellular calcium overload after an excessive CA surge could be attributed to cardiac contractile failure at the cellular level.^9^ CA is mostly produced by the chromaffin cells of the adrenal medulla and the postganglionic fibers of the sympathetic nervous system. More than a half century ago, by the use of fluorescent histochemical techniques, Glowinski and Iversen found that CA is distributed in complex systems of neurons almost everywhere in the brain, including the cerebellum.^10^ Snider et al^11^ found the cerebellar nuclei connect with a continuum of cells located on either side of the midline in the ventral tegmentum of the midbrain. By applying reduced-silver methods and horseradish peroxidase–treated materials in cat, Snider et al found evidence of a small Purkinje cell connection directly to the ventral tegmental area. As such, it is explainable that the rapid development of transient cardiac stunning right after acute large cerebellar stroke in our 4 patients could be due to a presumed CA surge triggered by acute stroke. Cardiac function returned to normal in 2 reported patients, as assessed by cardiac MRI or echocardiography 2 to 3 months after stroke onset (LVEF 57% 3 months later vs LVEF 26% on day 2 of acute stroke in case 1 and LVEF 55% 2 months later vs 37% at stroke onset in case 2, separately) without cardiac-specific therapy or intervention. LV noncompaction is a rare congenital cardiomyopathy that can affect both children and adults.^12^ Clinical symptoms in patients with LV noncompaction vary depending on disease severity and may be absent in asymptomatic individuals.^13^ One of our reported 4 patients (case 1) reported no cardiac disease at her baseline and denied palpitation, shortness of breath, or edema of distal extremities. She likely had poor cardiac reserve in the setting of asymptomatic LV noncompaction. When she developed acute cerebellar ischemic stroke, a CA storm was triggered by acute stroke that subsequently resulted in transient cardiac stunning. Cardiac MRI performed 3 months after acute stroke demonstrated that her cardiac function had returned to normal, with an LVEF of 57%. Our male patient (case 2) was healthy before his acute cerebellar stroke. His cardiac dysfunction was transient and resolved, returning to normal within 2 months of the onset of his stroke. Cardiac function was improved in just 5 days, with the LVEF increasing from the initial 15% to 50%, in our reported case 3. The fourth patient in this case series report demonstrated that the LVEF increased from the initial 15% to 30% in 5 days, as evaluated by TTE. In addition, her newly developed atrial fibrillation in the setting of a healthy baseline condition could be associated with her acute posterior circulation ischemic stroke, rather than being cardiogenic in nature.

CA excess not only seems to play a critical role in causing concurrent cardiac manifestations in the setting of acute cortical stroke involving the insular cortex^2^, ^3^, ^4^ but can also occur with acute cerebellar stroke in the absence of insular involvement. In addition, occult underlying cardiac pathology should be carefully investigated to identify potential pathogenetic mechanisms of transient cardiac stunning after brain insult. In this case series report, we illustrate that patients with acute ischemic cerebellar stroke can develop transient neurogenic cardiomyopathy without the need for cerebral cortical insult involving the insular cortex. This presumed neurogenic cardiac dysfunction resulting from acute ischemic stroke should have good recovery without permanent cardiac negative outcomes, in general.

## Funding Support and Author Disclosures

The authors have reported that they have no relationships relevant to the contents of this paper to disclose.
